# A Novel Interaction of *Nesidiocoris tenuis* (Hemiptera: Miridae) as a Biological Control Agent of *Bactericera cockerelli* (Hemiptera: Triozidae) in Potato

**DOI:** 10.3390/insects15040261

**Published:** 2024-04-11

**Authors:** Gabriela Esparza-Diaz, Raul T. Villanueva, Ismael E. Badillo-Vargas

**Affiliations:** 1 Texas A&M AgriLife Research and Extension Center, 2415 E. US Highway 83, Weslaco, TX 78596, USA; 2 Department of Entomology, University of Kentucky Research and Education Center, University of Kentucky, 348 University Drive, Princeton, KY 42445, USA; 3 Department of Entomology, Texas A&M University, 2475 TAMU, College Station, TX 77843, USA; 4 Biology Department, South Texas College, 400 N. Border, Weslaco, TX 78596, USA

**Keywords:** tomato bug, zoophytophagous, biological control agent, potato psyllid, Solanum tuberosum, zebra chip disease

## Abstract

**Simple Summary:**

The tomato bug is a generalist predator commonly used to control insect pests. This exotic mirid was found in 2012 in South Texas and has been established in this region. It was initially observed feeding on nymphs of the potato psyllid in tomato crops. The potato psyllid is the vector of the fastidious bacterium that causes disease in various night-shade crops, including potato zebra chip disease (ZC), with economic losses that by the mid-2000s escalated to tens of millions of dollars in the United States, Mexico, and Central America. We assessed interactions between tomato bugs and potato psyllids in three different environmental settings. First, we estimated the numeric response of tomato bugs preying on potato psyllids under laboratory and greenhouse conditions. Second, we evaluated the predator–prey interaction under controlled field cage conditions. Third, we exposed tomato bugs under controlled field release conditions to the natural occurrence of potato psyllids under a reduced insecticide program. Finally, we assessed its impact on ZC disease incidence, severity in potato tubers, and potato yield. In laboratory and greenhouse experiments, tomato bug response preying resulted in the potentially beneficial effects of the predacious tomato bug reducing potato psyllid populations. Overall, the controlled release of tomato bugs under field conditions significantly reduced potato psyllid incidence in potatoes. Furthermore, the combination of tomato bugs with a reduced insecticide program increased potato yields, but only reduced ZC tuber incidence in one of the two potato cultivars evaluated in one season. Findings from these studies indicate that tomato bugs could be effective as a biological control agent for potato psyllids in potato production.

**Abstract:**

*Nesidiocoris tenuis* (Hemiptera: Miridae) is a generalist predator commonly used to control the whitefly *Bemisia tabaci* in Europe. This mirid has been found and established in South Texas, where it was initially observed feeding on nymphs of the psyllid *Bactericera cockerelli* (Hemiptera: Triozidae) in open tomato fields. *B. cockerelli* is the vector of the fastidious bacterium “Candidatus Liberibacter solanacearum” that causes diseases in several solanaceous crops, including zebra chip (ZC) disease in potatoes. There is a need to better understand how this predator impacts the control of important crop pests, such as potato psyllids. We assessed the interactions between *N. tenuis* and *B. cockerelli* in three different environmental settings. First, we estimated the numeric response of *N. tenuis* preying on *B. cockerelli* under laboratory and greenhouse conditions. Second, we evaluated the predator–prey interaction under controlled field cage conditions. Then, we exposed *N. tenuis* under controlled field release conditions to the natural occurrence of *B. cockerelli*. Finally, we assessed the compatibility between the use of *N. tenuis* as a biological control agent in a field study and its impact on ZC disease incidence, severity in potato tubers, and potato yield. Laboratory and greenhouse experiments resulted in diverse types of functional model responses, including exponential and linear mathematical models. Our findings revealed a significant predation effect exerted by *N. tenuis*, resulting in a reduction of more than fourfold in the number of *B. cockerelli* nymphs per cage. Specifically, the nymphal population decreased from 21 ± 3.2 in the absence of *N. tenuis* to 5 ± 1.6 when *N. tenuis* was present. Furthermore, the combination of *N. tenuis* with a reduced insecticide program increased potato yields, but only reduced ZC tuber incidence in one of two potato cultivars evaluated, and in one season. Findings from these studies indicate that *N. tenuis* could be effective as a biological control agent for *B. cockerelli* in potato production in South Texas. This is the first report of *N. tenuis* preying on immature stages of any psyllid species.

## 1. Introduction

The tomato bug, *Nesidiocoris tenuis* (Reuter) (Hemiptera: Miridae), is a zoophytophagous insect originally found in Indonesia [1]. According to the Invasive Species Compendium [2], *N. tenuis* is present in North Africa, the Middle East, Japan, Australia, the Philippines, the Pacific Islands, North America, several countries in Asia [3], Cuba [4], Venezuela [5,6], Iran [7], Egypt [8], and India [9]. An introduction of *N. tenuis* was reported in 1987 in France [10], but there have also been invasions into new geographic areas beyond their natural habitats in Texas [11,12] and Mexico [13]. The successful colonization of new geographic areas by *N. tenuis* could be the result of rapid adaptations to environmental conditions favorable for its establishment [12]. *Nesidiocoris tenuis* is a voracious generalist predator frequently used in Europe to control the silverleaf whitefly (*Bemisia tabaci*, Hemiptera: Aleyrodidae), the greenhouse whitefly (*Trialeurodes vaporariorum* Hemiptera: Aleyrodidae) [14,15,16,17,18], and tomato pinworms (*Tuta absoluta*; Lepidoptera: Gelechiidae) [19].

The potato psyllid, *Bactericera cockerelli* (Šulc) (Hemiptera: Triozidae), is native to southern North America and is a key pest of solanaceous crops such as potatoes, tomatoes, eggplants, and peppers [20,21,22,23,24]. By the early 1900s, *B. cockerelli* had been recognized as having great invasive and harmful potential, predominantly in the western United States and Mexico [21,22,25,26,27,28]. Also, *B. cockerelli* has been reported in Guatemala [29], Honduras [30], Ecuador [31], New Zealand [32], Canada [33], and Australia [34]. Additional to the potential foliar damage referred to as “psyllid yellows” resulting from extensive and direct feeding by the psyllid nymphs [35,36,37,38,39,40,41], the most devastating effect associated with *B. cockerelli* is the transmission of the fastidious alpha-proteobacterium “*Candidatus* Liberibacter solanacearum” (Lso) to solanaceous plants [42,43,44]. This bacterial pathogen causes diseases in several solanaceous crops, including zebra chip (ZC), an economically important disease in potatoes in the United States, Mexico, and New Zealand [32,45,46,47,48,49]. Zebra chip disease has also been documented to occur in Guatemala, Honduras, El Salvador, and Nicaragua [45,50,51], and most recently in Ecuador [31]. The losses from ZC disease in potato cultivation were initially sporadic. However, by the mid-2000s, these losses escalated to tens of millions of dollars in the United States, Mexico, and Central America. This was primarily due to reduced yields and tuber quality, leading to a decline or complete loss of market value for the affected tubers [52]. ZC disease is responsible for millions of dollars in losses to potato producers and processors worldwide [43,48,49,53]. Symptoms of ZC disease are characterized by the production of tubers consisting of a zebra-like necrotic striped pattern [48,49,54]. Potato chips and French fries made from Lso-infected tubers are commercially unacceptable. Generally, tubers infected with Lso do not sprout, and if they do, produce hair sprouts or weak plants. Given the absence of a cure for Lso infection, ZC disease is currently managed through insecticide applications aimed at controlling *B. cockerelli* [48,49]. However, this management strategy is not sustainable, and recent studies have shown the development of insecticide resistance by certain populations of *B. cockerelli* in different parts of Texas [55]. Therefore, alternative management strategies are needed to effectively minimize losses caused by this insect pest and ZC.

**Figure 1:**
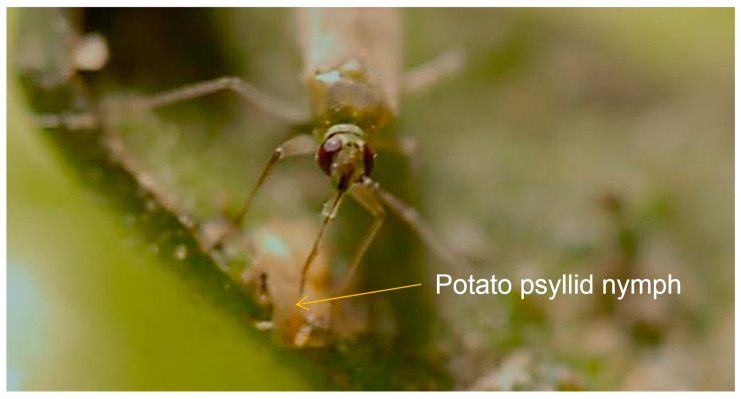
Spontaneous feeding of *Nesidiocoris tenuis* on a *Bactericera cockerelli* nymph on the sepal of tomato fruit from an organic field. Caption on Leica stereoscope S8APO, camera MC120HD (IN 56059), software Leica Application Suite (LAS19054; 0001408294), LAS Live Z Builder (912730457).

Since its discovery in southern Texas in 2012, the *N. tenuis* adult has been documented to feed on *B. cockerelli* nymphs in open organic tomato fields within the Lower Rio Grande Valley (LRGV) ecoregion, constituting the first observation of this behavior (Figure 1). The insect continued to thrive on local tomato crops during the winters of 2013, 2014, and 2015, clearly indicating that this exotic insect species had been established in South Texas [12]. These observations prompted us to investigate the potential of this recently established mirid predator in South Texas as a biological control agent of *B. cockerelli*. In this study, we explored the direct effect of the zoophagous feeding behavior of *N. tenuis* concerning changes in the population of *B. cockerelli* and any potential indirect impact on yield and reduction in symptoms associated with ZC in potatoes. The objectives of this study were to (1) describe the functional response of *N. tenuis* as a predator of *B. cockerelli* under laboratory and greenhouse conditions, (2) evaluate the predator and prey interaction under field cage conditions, (3) evaluate the use of controlled field releases of *N. tenuis* to natural occurrences of *B. cockerelli*, and (4) assess *N. tenuis* alone and in a reduced insecticide program to decrease ZC disease incidence, the severity on tubers, and effect on yields of two different potato cultivars (Atlantic and FL-1867).

## 2. Materials and Methods

### 2.1. Potato Psyllid Colony

Adult potato psyllids were initially collected using a hand-held portable vacuum aspirator from a pesticide-free potato field located at the Texas A&M AgriLife Research and Extension Center in Weslaco, TX (latitude 26.159244° and longitude −97.960701°). The insect collector was transported securely under controlled light and heat conditions within an insulated chamber. In 2014, a colony was established and maintained under laboratory conditions at a constant temperature of 24 °C, with 54% RH, and a 16:8 h photoperiod of artificial light intensity of 20.53 µmol/m^2^ s^1^. This colony was kept in an insect-rearing tent cage made of 60 × 60 × 60 cm nylon netting white mesh (MegaView Science Co., Ltd., Taichung City, Taiwan; BugDorm-2F120). The colony was infused every year with field-collected psyllids to maintain the fitness of the colony. Psyllids were identified as the Central haplotype, described by Swisher et al. [56]. To generate a Lso-infected colony, the psyllids were first established on Lso-infected tomato plants, and then 4th-instar nymphs were transferred onto Atlantic potato plants to synchronize the longevity and sexual maturity of infected adult psyllids. The proportion of total psyllids caught in traps in Texas was generally positive for Lso at ~3%, but the infection rate sometimes exceeded this value in certain fields and cultivation areas [53,57]. Although this proportion is small and decreases in newly emerged psyllids even on infected plants, at the end of the season the percentage of positive psyllids can reach up to 40% [58]. Before the trials, a sample of ten individual adult psyllids was tested monthly to confirm the presence of Lso in the insects using standard polymerase chain reaction PCR analysis [59].

### 2.2. Tomato Bug Colony

Adult *N. tenuis* collected from a tomato field in the LRGV were used to establish a colony under greenhouse conditions. Given *N. tenuis* ability to sustain its development exclusively on sesame (*Sesamum indicum* L. (Pedaliaceae)) [60], sesame was selected as the mirid’s host plant, as it can complete its life cycle solely by feeding on this plant. Sesame seeds used in the *N. tenuis* colony were pesticide-free. Sesame plants were cultivated in plastic trays and shielded from other insects and mites using a tent cage constructed from woven nylon netting mesh with a 160 µm aperture. Sesame plants were cultivated in a greenhouse with regulated temperature and humidity and were exposed to natural light. This colony was kept in an insect-rearing tent cage made of 60 × 60 × 60 cm polyester white mesh (MegaView Science Co., Ltd., Taichung City, Taiwan; BugDorm-2120) within a greenhouse. The tent cage had 144 sesame plants 40 cm tall. We renew 72 plants every week in each tent. The colony was maintained at 25 °C, with 59% RH, 16:8 h photoperiod, and a natural light intensity of 13.9 µmol/m^2^ s.

### 2.3. Tomato Bug Adults for Trials

Before the *N. tenuis* met *B. cockerelli*, their diet exclusively comprised sesame plants without any insect prey. Therefore, a zoophagous starvation period was not necessary for all trials. To control *N. tenuis* age, we used only mated individuals in equal numbers that were four days old and kept them under similar environmental conditions as mentioned above in the Tomato Bug Colony section.

### 2.4. Assessing the Model or Functional Response of N. tenuis Preying on B. cockerelli on Potato under Laboratory and Greenhouse Conditions

Although the most conventional method to determine the functional response is to expose an individual predator to different numbers of prey that are gradually increasing, here, we designed the experiment distinctly. In our studies, we exposed the same number of prey items to an increasing number of predators for the construction of the model or functional response relationship [61]. Exposing the same number of prey to an increasing number of predators helped answer the most important question during the establishment period of the arrival of *B. cockerelli* in potato fields: how many *N. tenuis* adults are required to control or reduce the size of the pest population in the field? For this, the relationship between *N. tenuis* adults and the *B. cockerelli* population was analyzed to estimate the number of predatory adults necessary to reduce the survival of both psyllid adults and their offspring (eggs and nymphs). To determine the math model for the predation or functional response of *N. tenuis*, different numbers of adults were kept in captivity with 4 d mated *B. cockerelli* couples on Lso-free Atlantic potato plantlets. Predator–prey ratios were used while holding the initial adult prey constant [62]. Then, four mating couples of *B. cockerelli* (four adult females and four adult males) were exposed to different numbers of *N. tenuis* adults (predator–prey ratio). Predator–prey ratios featured a combination of 2, 4, 6, 8, 10, or 12 *N. tenuis* adults with 4 of these mating couples of *B. cockerelli* for a total of six predator–prey ratios. Each of the six ratios was contrasted with a single control group, which consisted of the same number of *B. cockerelli* mating couples without *N. tenuis* present (0 *N. tenuis*). Every four sets of pairs of 4-day-old and mated *B. cockerelli* couples were enclosed on Lso-free plantlets using an inverted PET Cup (591 mL Clear Straight Wall #TN20 SOLO^®^, Lake Forest, IL, USA) in the laboratory, while white organza bags (15.24 × 22.86 cm WLEAD) were used in the greenhouse. An opening (5 × 5 cm) was cut out from the bottom of each plastic cup and covered with organza mesh to ventilate the environment experienced by the enclosed insects in the laboratory. The predator–prey ratio was formed as the initial number of *N. tenuis* adults per *B. cockerelli* adult. When the enclosure was ready, the respective number of *N. tenuis* per predator–prey ratio was placed immediately into the enclosures for predation tests. Each predator–prey ratio was replicated five and six times under laboratory and greenhouse conditions, respectively. A HOBO^®^ U12-012 data logger (Forestry Suppliers Inc., Jackson, MS, USA) was set in the laboratory and greenhouse to record the temperature and relative humidity every 24 h for a total of 216 h. The laboratory and greenhouse conditions had a photoperiod of 16:8 (L:D) and 13:11 (L:D), respectively. We followed the predatory activity of adult *N. tenuis* in *B. cockerelli* nymphs 10 d after the initiation of the predation tests. The total prey population of *B. cockerelli* was recorded as the number of eggs, nymphs, and adults alive per predator–prey ratio.

### 2.5. Efficacy of N. tenuis on B. cockerelli in Potato in Field Cage Studies

Lso-free potato seeds were planted in field cages on 18 December 2015 and 22 December 2016 for the growing seasons 2016 and 2017 field studies, respectively. The cages were established in sandy clay loam soil in a research field at the Texas A&M AgriLife Research and Extension Center in Weslaco, TX. Individual Atlantic potato plants were enclosed in BugDorm^®^ insect tents (MegaView Science Co., Ltd., Taichung City, Taiwan; soil emergence trap-headless insect regarding tent, 60 × 60 × 60 cm, catalog number BT2007) at planting. Drip tape irrigation was used to water the plants in the cages. Ten adult 4-day-olds of *N. tenuis* and *B. cockerelli* each were placed on the caged potato plant. A caged potato plant infested with ten *B. cockerelli* adults (sex ratio 1:1) without predators was used as a control treatment. Both treatments were replicated five times each during each growing season. The predatory activity of adult *N. tenuis* (sex ratio 1:1) on *B. cockerelli* was assessed by counting the number of *B. cockerelli* eggs, nymphs, and adults present in each predator–prey ratio cage, under a stereoscope (Leica S8APO, Leica Microsystems Inc., Deerfield, IL, US) on leaflets. This evaluation was conducted ten days following the initiation of the predation tests. Additionally, the numbers of *N. tenuis* individuals were recorded in all cages under the stereoscope (Leica S8APO). Furthermore, we estimated the proportions of *N. tenuis* adults found alive ten days after the initiation of the experiment concerning the number of adults originally introduced at the beginning of the trial. The population metric of *B. cockerelli* (number of eggs and nymphs per cage) was the dependent variable, while the treatment condition (with or without the predator) served as the independent variable, observed ten days after the commencement of predation tests.

### 2.6. Predation Efficacy of N. tenuis on B. cockerelli on Potato under Field Conditions, Coupled with a Reduced Insecticide Program, and the Impact on Potato Yield and Incidence and Severity of Zebra Chip Disease

The field study assessing the impact of *N. tenuis* on natural populations of *B. cockerelli* was carried out in three treatments and an untreated control. We used only releases of *N. tenuis* as a biological agent with and without applications of insecticides in an alternate plan to control the natural populations of *B. cockerelli*. Also, we applied this insecticide regimen (reduced insecticide program: RIP) without the biological control agent. All of them were compared with the untreated control. The four treatments consisted of (1) the release of *N. tenuis* and the reduced insecticide program (Treatment: *N. tenuis* + RIP), (2) the reduced insecticide program alone (Treatment: RIP), (3) the release of *N. tenuis* (Treatment: *N. tenuis)*, and (4) the untreated control. Our reduced insecticide program consisted of three and four applications of insecticides per season for 2016 and 2017, respectively (Table 1). The treatments were conducted in an experimental field of the Research and Extension Center in Weslaco, Texas, and potato plots were planted with two cultivars, Atlantic and FL-1867. The untreated control field was in an organic area. Lso-free seed tubers of the Atlantic and FL-1867 cultivars each were planted on 18 December 2015, and 22 December 2016, and grown in the same field during the growing seasons in 2016 and 2017, respectively. Tubers were planted every 45 cm between plants, and 72.6 cm between rows. To shelter *N. tenuis* populations, two rows of insecticide-free sesame seeds were planted along both sides of the experimental potato plot as an alternative food source just in the *N. tenuis* treatment and the *N. tenuis*+ RIP treatment during this trial. Each plot consisted of four 35 m long rows with 300 plants per variety divided into four replications. Each experimental unit consisted of 75 plants. Plots had two 1.5 m sorghum rows that served as windrows to prevent insecticide drift. We used a clumped segregation design to avoid biocontrol agent cross-contamination between the treatments. Treatments were physically separated from each other by 300 m of uncultivated area.

#### 2.6.1. Controlled of *N. tenuis* Releases

The controlled *N. tenuis* releases (sex ratio 1:1) were conducted using a 16 oz Mini Mosquito Breeder without a lid (1425DG Bioquip, Rancho Dominguez, CA, USA). This *N. tenuis’* reservoir was fixed was attached to a pole placed in the center of the experimental potato plot for the corresponding treatments. As the first study of *N. tenuis* feeding on *B. cockerelli* under field conditions, we released a single *N. tenuis* at a rate of only one adult per plant for the effective biological control of naturally occurring *B. cockerelli* [63]. The controlled releases of *N. tenuis* adults and the insecticide applications were conducted when the wind speed was less than 12.5 Km/hand rain was absent.

#### 2.6.2. Scheduled-Program Controlled Releases of *N. tenuis*

Controlled releases of *N. tenuis* adults were conducted from 4 February to 28 March 2016 and from 20 January to 7 April 2017 (Table 1). Predation-efficacy studies under open-field conditions were recorded weekly during the 2016 and 2017 potato-growing seasons from December 2015 to April 2016 and December 2016 to April 2017, respectively.

#### 2.6.3. Scheduled Program for the RIP

The scheduled program for the RIP is shown in Table 1 for the 2016 and 2017 trails. Insecticide applications in the RIP were spaced to allow for releases of *N. tenuis* and to promote its predation on *B. cockerelli*. The RIP consisted of spirotetramat, abamectin, and pymetrozine, for 2016 and 2017, while spinetoram was added in 2017 (Table 1). All these insecticides have a different mode of action and showed proven efficacy against *B. cockerelli* [64,65] as well as compatibility with *N. tenuis*. According to previous reports, spirotetramat was compatible with *N. tenuis* [66]; likewise, abamectin was classified as slightly harmful, and pymetrozine was not toxic to *N. tenuis* [67], although it could decrease its feeding rate [68].

#### 2.6.4. Insecticide Applications and Organic Management

For foliar insecticide applications, a spider sprayer using a CO_2_ pump was used. The spray system for each row consisted of a central flat spray nozzle for the top of the potato plant and two lateral hollow cones for a full spray of the plant sides. The insecticide solution for total crop coverage was sprayed at a rate of 355 L/ha and a pressure of 3 kg/cm^2^. The sprayer system was calibrated for a homogeneous spray and an equal flow rate per nozzle before the applications to comply with the commercially recommended label rates Additionally, we sprayed a preventive application of fungicide without side effects on *N. tenuis* [69] comprising a clarified hydrophobic extract of neem oil (70% concentration) at three ZMA plus copper octanoate (copper soap 10%) at 2% *v*/*v*. Foliar fertilization was made during the vegetative crop stage with 1% of fulvic acid at 16 ZM to gal/acre.

#### 2.6.5. Population Dynamics of *B. cockerelli* and Predator

In all the treatments, the population dynamics of *B. cockerelli* were monitored during all the experiments. Leaf sampling was carried out from 2 February to 1 April 2016, and from 25 January to 13 April 2017. The numbers of eggs and nymphs of *B. cockerelli* per compound leaf per plant were recorded, and a leaf was taken from the middle plant canopy. Twenty leaves were collected from random plants of the two middle rows of each experimental plot for both potato cultivars per sampling date. A compound potato leaf consisted of eight leaflets (a terminal leaflet at the distal end of the leaf and seven lateral leaflets); however, the number of lateral leaflets could vary greatly, from two to at least 18 and probably more. Leaves were placed in plastic bags, placed in a cooler, and transported to the laboratory, where the specimens were counted using a stereoscopic microscope. The response variable was the number of eggs plus nymphs per compound leaf (quantified by the number of *B. cockerelli* immatures/leaf). The explanatory variable consisted of a four-level treatment, which included a reduced insect program without the biological control agent (RIP), along with an untreated control. In 2016 and 2017, we sampled a total of 580 and 960 compound potato leaves per potato variety, respectively.

The suggested economic threshold for *B. cockerelli* adults is three per sticky trap [70]. However, there is no established economic population threshold for *B. cockerelli*, necessitating control measures upon detection of the pest in the field [71]. Additionally, in the Lower Rio Grande Valley (LRGV), the early control of *B. cockerelli* is essential due to the presence of psyllids at the early stage of plant emergence [48]. *Bactericera cockerelli* adults were monitored at the same frequency by yellow sticky traps and foliar sampling. Insect monitoring with yellow sticky traps has proven to be effective and provided a high probability of *B. cockerelli* detection under open-field trials [72]. Before any treatment, the natural colonization of *B. cockerelli* in the plots was determined with an initial count of adult populations by trapping and immature stages in leaves. The natural colonization of *N. tenuis* and *B. cockerelli* adults was detected with yellow sticky traps and by counting immature stages of *B. cockerelli* on potato leaves in the insecticide-free plots and without *N. tenuis* release.

The monitoring of adult *B. cockerelli* was achieved using a yellow sticky card per replication at one card per 80 plants. The card was placed in the middle of the plot in the two central potato rows. Tallies of *B. cockerelli* on sticky yellow traps were recorded using a stereomicroscope Leica S8APO. Monitoring in traps was carried out from 1 January to 25 March 2016, and from 1 January to 13 April 2017. The response variable was the population of *B. cockerelli* measured as adults per trap. The explanatory variable consisted of a four-level treatment, which included a reduced insect program without the biological control agent (RIP), along with an untreated control. In 2016 and 2017, we sampled a total of 128 and 220 traps per potato variety, respectively.

#### 2.6.6. Impact on Potato Yield and Incidence and Severity of Zebra Chip Disease

All tubers of the two central rows of each experimental plot were harvested to estimate tuber yield (g/plant) from 20 plants, from which a sample of 20 tubers selected randomly was taken to estimate the percentage of tubers exhibiting ZC symptoms. Tubers were harvested on 22 April 2016, and 27 April 2017. Tuber weight was estimated per plant. ZC incidence in fresh tubers was estimated by cutting a latitudinal and central slice per tuber with an electric slicer calibrated at ~0.1 mm thickness (Rival Fold-Up Food Slicer White Model MS1043-W, TerraceKansas City, MO, USA) and assessing the disease vascular discoloration symptoms. Zebra chip symptoms in fried potatoes were recorded after deep-frying the sliced chips in canola oil for 3 min at 191 °C in a commercial fryer, as described by Munyaneza et al. [43]. The rates of ZC were recorded on 20 random tubers per plot.

### 2.7. Data Analysis

The predator–prey ratio was the independent variable, and the total prey population was the dependent variable. Regression analysis was used to look at the relationship of total prey populations with predator–prey ratios. Subsequently, this relationship was interpreted according to the three models proposed by Holling [73]. As a result, mathematical models of functional responses to the impact of *N. tenuis* on the *B. cockerelli* population were obtained. The total population of *B. cockerelli* as $\bar{x}$ ± and $\bar{x}$ ± CI was plotted as a function of the adult prey–predator ratios under laboratory and greenhouse conditions, respectively. The analysis of the mathematical model of response to predation was performed with Statistica™ (Version 113.5.0.17, 2018 Edition: StatSoft Inc., Tulsa, OK, USA).

Insect population data obtained from both open-field and cage studies were transformed using the square root (x + 0.5, where x is the mean of insect population) before analysis. Data were transformed with the square root to achieve homoscedasticity in the statistical model to test the effect of factors on population abundance [74].

For the field study, we employed Clustered Segregation as the experimental design, incorporating a pseudo replication [75]. This approach necessitated an unconventional statistical analysis [76]. We used the Mixed Model (SAS Institute 2011). By each potato variety in two seasons, we examined the influence of treatment (with and without *N. tenuis* releases, RIP, and RIP plus *N. tenuis*) on *B. cockerelli* populations (by leaf and trap), the percentage of ZC detected in tubers, and yield tuber per plant. The SAS MIXED procedure uses a restricted maximum likelihood (REML) estimate. The effective degrees of freedom for the fit model were estimated using the Containment method. The model included a fixed effect of treatment and experimental replication was a random factor, each with four levels. The variable repeated was the sampling date for the *B. cockerelli* populations per leaf or trap, with eight and 12 levels in 2016 and 2017, respectively, except for 13 levels for this population on traps in 2017. We used the type-3 sums of the squares test for significant interaction terms. If the main effect or effect interaction was found to be significant, treatments were compared using the Tukey–Kramer multiple comparisons adjustment to identify pairwise differences (α < 0.05). Statistical analysis was performed with SAS (version 9.4, Cary, NC, USA. SA Institute Inc.). Graphs were all performed with Statistica™ (Version 113.5.0.17, 2018 Edition: StatSoft Inc., Tulsa, OK, USA). Non-transformed means and standard errors are presented on tables and graphs.

## 3. Results

### 3.1. Assessing the Mathematical Model and Functional Response of N. tenuis Preying on B. cockerelli on Potato under Laboratory and Greenhouse Conditions

**Figure 2:**
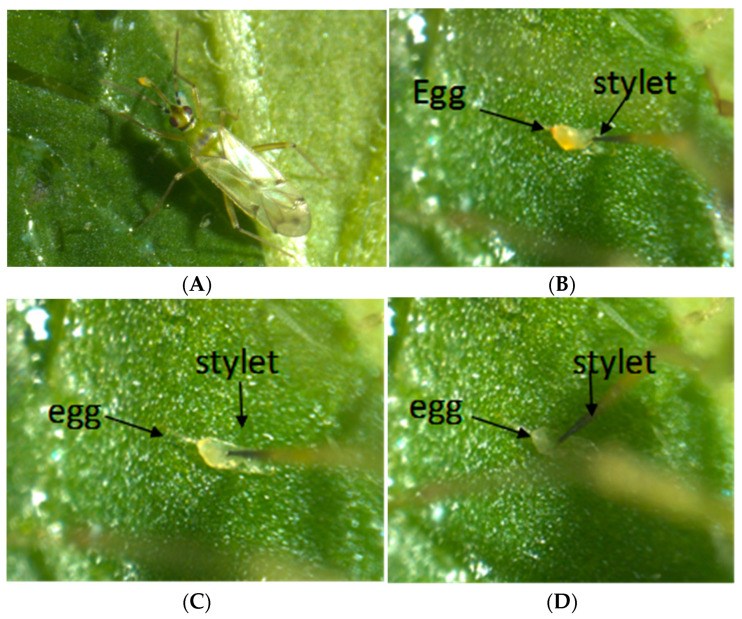
*Nesidiocoris tenuis* feeding sequence: (**A**) An adult exploring a *Bactericera cockerelli* egg. (**B**–**D**) a zoomed sequence view showing the stylet of *N. tenuis* feeding on the same egg until the second panel. Figures should be placed in the main text near the first time they are cited. Captions on Leica stereoscope S8APO, camera MC120HD (IN 56059), software Leica Application Suite 4.0 (LAS19054; 0001408294), LAS Live Z Builder (912730457).

**Figure 3:**
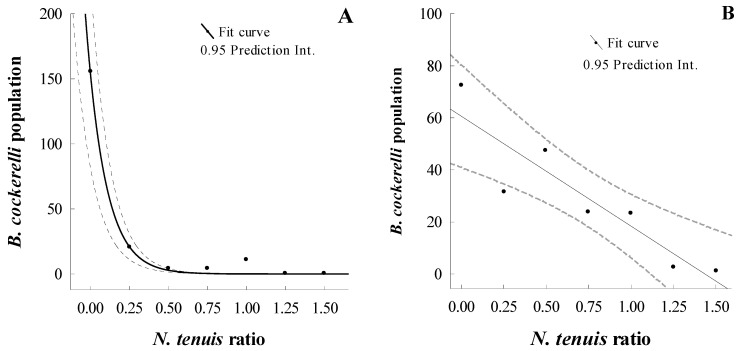
Math-model curves of *Nesidiocoris tenuis* and *Bactericera cockerelli* under (**A**) laboratory and (**B**) greenhouse conditions. Black dots are the mean population of *B. cockerelli*.

We observed an adult *N. tenuis* preying on a *B. cockerelli* egg and collected graphic evidence until the internal fluid of the egg was consumed, which indicated that *N. tenuis* was capable of successfully feeding on *B. cockerelli* eggs (Figure 2A–D). The functional response of *N. tenuis* preying upon *B. cockerelli* under laboratory and greenhouse conditions was determined in this study. Under laboratory and greenhouse conditions, the results showed that *B. cockerelli* numbers declined as the predator–prey ratio increased. The highest decrease in *B. cockerelli* abundance was reached with a prey—predator ratio of 0.5 under laboratory conditions, while under greenhouse conditions this ratio was 1.5 (three times greater than in the laboratory) (Figure 3A and Figure 3B, respectively). These data implied two distinct mathematical models for the laboratory and greenhouse (Figure 3). The model for the laboratory study was an exponential function response type III: *y* = *a* × *e*^(−*b* × *x*)^; where *y*: *B. cockerelli* population (eggs, nymphs, and adults alive); *a*: constant = 156.15 ± 22.94; *b*: constant −8.02 ± 4.74; and *x*: *N. tenuis* ratio (number of *N. tenuis* adult per *B. cockerelli* adult). The model for the greenhouse study was a linear type I: *y* = *a* − *bx*; where *y* = *B. cockerelli* population (eggs, nymphs, and adults alive); *a*: constant = −0.5; *b*: constant = −42.26 ± 10.84; and x: *N. tenuis* ratio (number of *N. tenuis* adult per *B. cockerelli* adult).

### 3.2. Efficacy of N. tenuis on B. cockerelli in Potato in Field Cage Studies

**Figure 4:**
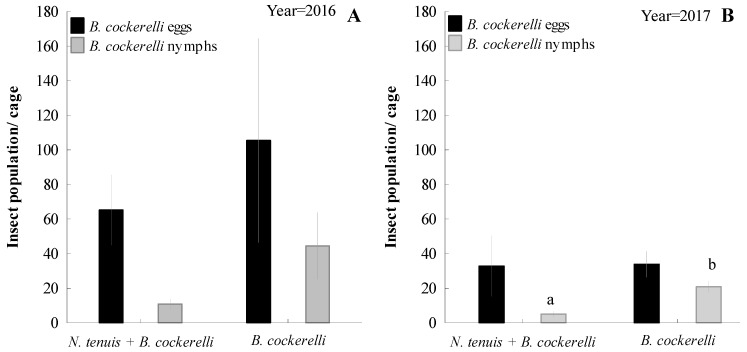
Effect of *Nesidiocoris tenuis* on the mean population of *Bactericera cockerelli* (±SEM) eggs and nymphs estimated per cage in experimental field studies under controlled field conditions (caged plant) during 2016 (**A**) and 2017 (**B**) potato-growing seasons. Different lower-case letters at the top of the columns show significant differences for that life stage between treatments (*p* < 0.05, Fisher’s LSD after an ANOVA test).

In both the 2016 and 2017 seasons, the numbers of the eggs and nymphs of *B. cockerelli* decreased in the presence of *N. tenuis* (Figure 4). However, the results showed a significant effect of *N. tenuis* as a predator of *B. cockerelli* nymphs with 5 ± 1.6 nymph/cage ($\bar{x}$ ± SEM) compared to *B. cockerelli* alone (21 ± 3.2 nymph/cage $\bar{x}$ ± SEM; F = 18.85; df = 1, 18; *P* = 0.002) in 2017. *Nesidiocoris tenuis* showed a nymphal production of 0.4 ± 0.24 and 0.4 ± 0.4 nymph/cage ($\bar{x}$ ± SEM) for 2016 and 2017 under field conditions, respectively. *Nesidiocoris tenuis* was able to reduce *B. cockerelli* populations when compared with cages without *N. tenuis*.

### 3.3. Predation Efficacy of N. tenuis on B. cockerelli on Potato under Field Conditions, Coupled with a Reduced Insecticide Program, and the Impact on Potato Yield and Incidence and Severity of Zebra Chip Disease

**Figure 5:**
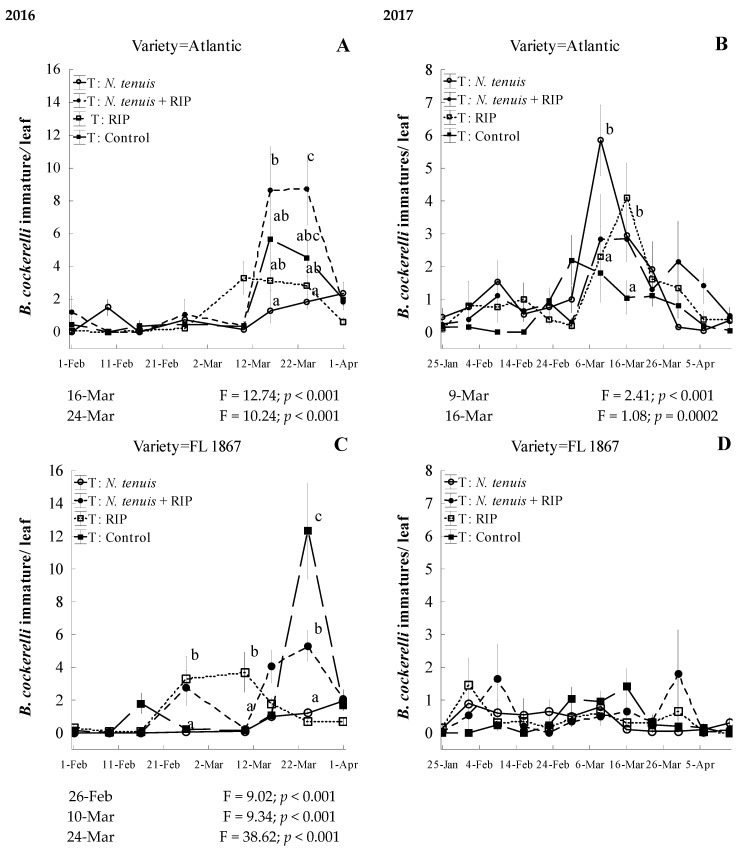
Effect of treatments on mean populations of immature stages of *Bactericera cockerelli* (±SEM) on two potato cultivars, Atlantic and FL 1867, in experimental studies under open-field conditions during the 2016 (**A** and **B**, respectively) and 2017 (**C** and **D**, respectively) potato-growing seasons. Different lower-case letters show significant differences in *B. cockerelli* populations between treatments on dates shown under the graphs by multiple paired comparisons (Tukey–Kramer, *p* < 0.05).

**Figure 6:**
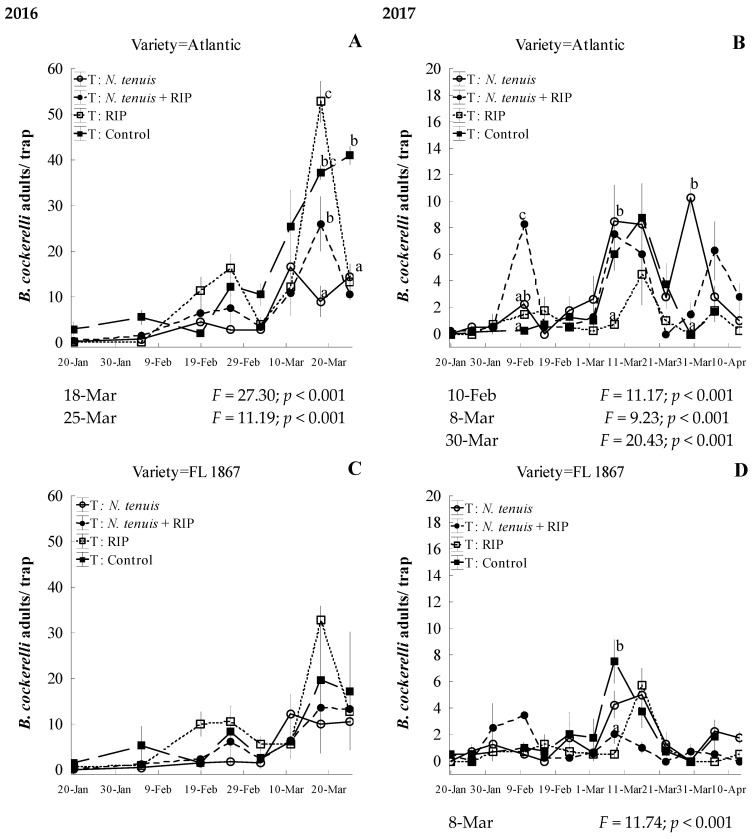
Effect of treatments on the mean population of *Bactericera cockerelli* adults (±SEM) on two potato cultivars, Atlantic and FL 1867, in experimental studies under open-field conditions during the 2016 (**A** and **B**, respectively) and 2017 (**C** and **D**, respectively) potato-growing seasons. Different lower-case letters indicate significant differences in *B. cockerelli* populations between treatments on dates shown under the graph by multiple paired comparisons (Tukey–Kramer HSD, *p* < 0.05).

The population of *B. cockerelli* naturally fluctuated in most of the treatments during the study (Figure 5A–D and Figure 6A–D). A total of 0.7–0.9 *N. tenuis* adults per plant were released during the growing season in plots with or without RIP during the field study in the Atlantic or FL-1867 cultivars.

During the 2016 field trial with Atlantic potatoes, there were significant date (F = 224.62; df = 7, 545; *p* < 0.001), treatment (F = 3.71; df = 3, 545; *p* = 0.011), and date–treatment interactions (F = 224.62; df = 21, 545; *p* < 0.001) on *B. cockerelli* immatures per leaf. The *N. tenuis* treatment had a significant reduction in immature *B. cockerelli* stages on two dates, 16 and 24 March 2016 (Figure 5A). This treatment significantly reached the lowest *B. cockerelli* immature numbers of 1.3 ± 0.4 ($\bar{x}$ ± SEM) per leaf on 16 March 2016. On this date, the treatments reached 3.2 ± 0.6; 5.7 ± 5.1; and 8.7 ± 2.7 *B. cockerelli* immature per leaf ($\bar{x}$ ± SEM) in the RIP, control, and *N. tenuis* + RIP, respectively (Figure 5A).

In the 2017 Atlantic cultivar, date, treatments, and date– treatment interactions significantly influenced the numbers of *B. cockerelli* immatures per leaf: (F = 15.37; df = 11, 909; *p* < 0.001), F = 4.22; df = 3, 909; *p* = 0.005), and (F = 2.68; df = 33, 909; *p* < 0.001), respectively. The *N. tenuis* treatment was the only one that did not significantly reduce the immature stages of *B. cockerelli* on 9 March, while RIP, *N. tenuis* + RIP, and control treatments had a similar number of *B. cockerelli* immatures per leaf (Figure 5B). On this date, treatments reached *B. cockerelli* immatures per leaf ($\bar{x}$ ± SEM) of 1.8 ± 0.9, 2.3 ± 0.9, 2.9 ± 1.3, 5.9 ± 1.1 *B. cockerelli* in the control, RIP, *N. tenuis* + RIP, and *N. tenuis*, respectively (Figure 5B), whereas, on 16 March, all treatments had significantly higher numbers of *B. cockerelli* immature per leaf than the control (Figure 5B). On this date, treatments reached *B. cockerelli* immature per leaf ($\bar{x}$ ± SEM) of 1.1 ± 0.5, 2.9 ± 0.7, 3.0 ± 0.6, and 4.1 ± 1.1 in the control, *N. tenuis* + RIP, *N. tenuis*, and RIP, respectively (Figure 5B).

During the 2016 field trial with FL-1867 potatoes (Figure 5C), there were significant differences in *B. cockerelli* immature per leaf in dates (F = 23.93; df = 7, 545; *p* < 0.001), treatments (F = 7.35; df = 3, 545; *p* < 0.001), and date– treatment interactions (F = 8.40; df = 21, 545; *p* < 0.001). There were significant differences between treatments on 26 February, 10 and 24 March (Figure 5C). On 24 March 2016, the peak infestation of *B. cockerelli* was observed under the control treatment when in the *N. tenuis* and RIP treatments significantly impacted the immature stages. On this date, the RIP treatment and *N. tenuis* had 0.7 ± 0.2 and 1.2 ± 0.3 *B. cockerelli* immature per leaf ($\bar{x}$ ± SEM; Figure 5C), respectively. The two other treatments increased prey numbers, reaching their maximum of the season with 12.3 ± 2.9 and 5.3 ± 0.9 of *B. cockerelli* immature per leaf ($\bar{x}$ ± SEM) in the control and *N. tenuis* + RIP, respectively (Figure 5C).

In the 2017 FL-1867 field trial, there was a significant effect of date (F = 3.6; df = 11, 909; *p* < 0.001) and of an interaction effect between date and treatment (F = 1.68; df = 33, 909; *p* = 0.009) on *B. cockerelli* immatures per leaf. However, there was no significant effect of treatment (F = 0.1; df = 33, 909; *p* = 0.9594) on *B. cockerelli* immatures per leaf. Although there was a significant date-treatment interaction by mixed fixed repeated measures analysis, significant differences pairwise were not possible to determine (Tukey–Kramer Adj. *p* > 0.05; Figure 5D). In this season, these results were not conclusive, since prey numbers were extremely low. The highest peak population was 1.8 ± 1.3 of *B. cockerelli* immature/leaf ($\bar{x}$ ± SEM) of *N. tenuis* + RIP treatment.

Results on the number of adult populations in the 2016 Atlantic potato variety trial showed a significant effect on *B. cockerelli* adult/trap of date (F = 39.15; df = 7, 93; *p* < 0.001), treatment (F = 21.03; df = 3, 93; *p* < 0.001), and interaction effects of date–treatment (F = 5.07; df = 21, 93; *p* < 0.001). The analysis showed that the RIP treatment reached a significantly higher number of adult preys per trap ($\bar{x}$ ± SEM) on 18 March with 52.7 ± 4.5, while *N. tenuis* had a decrease to a significant lowest number with 9.0 ± 3.4 of *B. cockerelli* adults per trap ($\bar{x}$ ± SEM; Figure 6A). In the other two treatments, the increase in prey numbers were reaching 37.3 ± 1.5 and 26.0 ± 6.0 *B. cockerelli* adults per trap ($\bar{x}$ ± SEM) in the control and *N. tenuis* + RIP, respectively (Figure 6A). On March 25, all treatments significantly reduced adult *B. cockerelli* per trap, and the control had its highest infestation of the season (41.0 ± 2.0 of *B. cockerelli* adults per trap $\bar{x}$ ± SEM; Figure 6A).

In the Atlantic variety on the field trail, we observed a significant date effect (F = 17.64; df =12, 167; *p* < 0.001), treatment (F = 10.82; df = 3, 167; *p* < 0.001), and date–treatment interaction (F = 4.04; df = 34, 167; *p* < 0.001) on *B. cockerelli* adults per trap in the 2017 season. At the beginning of the season on 10 February, only *N. tenuis* + RIP treatment had a significantly high population of *B. cockerelli* adults per trap (8.3 ± 0.5 $\bar{x}$ ± SEM; Figure 6B). On 8 March, RIP significantly reduced *B. cockerelli* adults per trap to 0.8 ± 0.3 ($\bar{x}$ ± SEM; Figure 6B). At the end of the season on 30 March, the *N. tenuis* treatment had a significantly higher population of *B. cockerelli* adults per trap (10.3 ± 1.1 $\bar{x}$ ± SEM; Figure 6B). The RIP treatment kept significantly lower numbers on these three dates.

In the 2016 FL-1867 trial, there was a significant effect of date (F = 18.32; df = 7, 93; *p* < 0.001) and treatment (F = 4.29; df = 3, 93; *p* = 0.007), but not of date–treatment interactions (Figure 6C) on *B. cockerelli* adults per trap. Across the dates, results indicated that *N. tenuis* and *N. tenuis* + RIP had significantly lower numbers of *B. cockerelli* adults per trap (4.8 ± 1.0 and 5.7 ± 1.0 $\bar{x}$ ± SEM, respectively), followed by the control and RIP treatments (7.8 ± 2.7 and 9.9 ± 1.9 $\bar{x}$ ± SEM, correspondingly).

In the FL-1867 trial in 2017, there was a significant main effect of date (F = 10.48; df = 12, 167; *p* < 0.001), treatment (F = 4.61; df = 3, 167; *p* = 0.004), and the interaction effect between date and treatment (F = 2.56; df = 34, 167; *p* < 0.001) on *B. cockerelli* adults per trap. In the middle of the season on March 8, the maximum population reached 7.5 ± 1.7 *B. cockerelli* adults per trap ($\bar{x}$ ± SEM) in the treatment of control while the other treatments were found with significantly lower numbers, with 4.3 ± 1.0, 2.0 ± 0.7, and 0.5 ± 0.3 ($\bar{x}$ ± SEM) of *N. tenuis*, *N. tenuis* + RIP, and RIP treatments, respectively (Figure 6D).

We followed the nymph-predator populations. In the Atlantic trial in 2016, results showed no significant main effect of treatment (F = 0.89; df = 5, 535; *p* > 0.05), date (F = 0.68; df = 3, 535; *p* > 0.05), and the interaction of date–treatment (F = 1.10; df = 21, 535; *p* > 0.05) on *N. tenuis* nymphs/leaf. In the same season, FL 1867 trail showed no significant main effect of treatment (F = 1.63; df = 3, 535; *p* > 0.05), date (F = 1.05; df = 7, 535; *p* > 0.05), and interaction (F = 0.87; df = 21, 535; *p* > 0.05) on *N. tenuis* nymphs/leaf. In 2017, the Atlantic trial showed no significant main effect of treatment (F = 0.82; df = 11, 909; *p* > 0.05), but a significant effect of date (F = 3.06; df = 3, 909; *p* < 0.001), and no significant effect for the date–treatment interaction (F = 1.10; df = 33, 909; *p* > 0.05) on *N. tenuis* nymph/leaf. At the beginning of the 2017 season, the Atlantic trail had a peak of *N. tenuis* nymphs/leaf (0.1 ± 0.05 $\bar{x}$ ± SEM). In the same season, the FL 1867 trail showed no significant effect of treatment (F = 0.86; df = 7, 93; *p* > 0.05), date (F = 0.67; df = 3, 93; *p* > 0.05), and date–treatment interaction (F = 1.05; df = 21, 93; *p* > 0.05) on *N. tenuis* nymph per leaf. The extremely low natural occurrence of *N. tenuis* nymphs/leaf on the control was 0.02 ± 0.01 ($\bar{x}$ ± SEM) in the 2017 season, and nymphs were untraceable in 2016. Even when controlled releases of adults were carried out, also nymphal presence was exceptionally low in the *N. tenuis* treatment, with 0.04 ± 0.01 ($\bar{x}$ ± SEM) of *N. tenuis* nymphs/leaf in 2016. Likewise, with *N. tenuis* + RIP treatment, few *N. tenuis* nymphs/leaf were observed with values of 0.05 ± 0.03 and 0.02 ± 0.01 ($\bar{x}$ ± SEM) in 2016 and 2017, respectively.

Likewise, we followed a population of *N. tenuis* adults in sticky traps. Only the Atlantic trial in 2016 showed a significant effect of treatment (F = 3.31; df = 3, 167; *p* = 0.021), date (F = 2.86; df = 12, 167; *p* = 0.001), and date–treatment interaction (F = 3.21; df = 34, 167; *p* < 0.001) on *N. tenuis* adults/trap. Only the *N. tenuis* treatment had significantly higher numbers of *N. tenuis* adults per trap (0.25 ± 0.25 $\bar{x}$ ± SEM) on 26 February 2016 (F = 37.29; *p* < 0.001) and above zero on treatments with *N. tenuis* + RIP, RIP, and the untreated control. We had evidence that released *N. tenuis* adults were not significantly trapped in the yellow sticky traps. Only 1: 290 *N. tenuis* adults released were recaptured on the traps. These results showed that two rows of sesame without insecticide had no significant difference in the population of *N. tenuis* even though they were for a refuge of populations of *N. tenuis* in treatments with releases of these predators.

**Figure 7:**
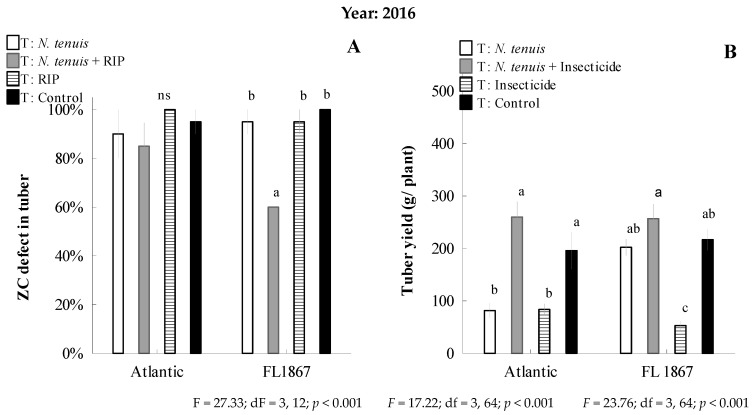

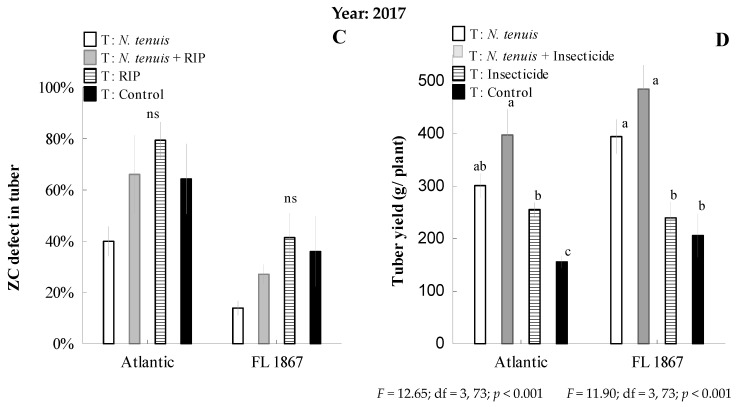
The estimated incidence of ZC disease in tubers (% ± SEM) and potato yield per plant (±SEM) by potato cultivar in experimental studies under open-field conditions during 2016 (**A** and **C**, respectively) and 2017 (**B** and **D**, respectively) potato-growing seasons. Zebra chip defect in tuber and yield per plant between treatments per potato cultivar not connected by the same letter are significantly different using multiple pairwise comparisons (Tukey–Kramer HSD, *p* < 0.05), and “ns” shows non-significant (*p* > 0.05). RIP = reduced insecticide program.

Regarding the incidence of ZC disease in 2016, there was a significant main effect of treatment. We only observed a significant reduction in the percentage of infected FL-1867 tubers in the treatment with *N. tenuis* + RIP, where 60% ± 0.0 ($\bar{x}$ ± SEM) of the tubers were infected (Figure 7A). In 2017, there was no significant difference in tuber infection, and the percentage of tubers with ZC symptoms was estimated at 14 ± 3% and 40 ± 6% ($\bar{x}$ ± SEM) when *N. tenuis* was released on FL-1867 and Atlantic potato plants, respectively (Figure 7C).

In 2016, there was a significant main effect of treatment on yield tuber per plant and for Atlantic and FL 1867, respectively. Only the *N. tenuis* + RIP treatment in the Atlantic and FL-1867 cultivars was like the control treatment in yield per plant, with 260 ± 29 and 257 ± 28 g/plant ($\bar{x}$ ± SEM; Figure 7B), respectively. Also, the *N. tenuis* treatment was like the control on the FL 1867 variety, with 227 ± 15 g/plant ($\bar{x}$ ± SEM; Figure 7B).

In 2017, There was a significant main effect of treatment on yield tuber per plant and for Atlantic and FL 1867, respectively. The *N. tenuis* + RIP trend continued in 2017, and this treatment increased the yield significantly more than the control, reaching 398 ± 49 and 485 ± 46 g/plant ($\bar{x}$ ± SEM) for both cultivars, Atlantic and FL1867, correspondingly (Figure 7D). Also, the *N. tenuis* treatment was like *N. tenuis* + RIP, with 395 ± 32 g/plant for the FL 1867 variety ($\bar{x}$ ± SEM; Figure 7D).

## 4. Discussion

We elucidate the predatory role of *N. tenuis* in controlling *B. cockerelli* infestation in potatoes. We showed evidence of the spontaneous predation of *B. cockerelli* nymph by *N. tenuis* in the LRGV. Also, we observed *N. tenuis* preying upon eggs of *B. cockerelli* on potato plants under laboratory conditions. Under controlled laboratory and greenhouse conditions, the predator–prey ratio vs. prey abundance resulted in exponential (type III; [73]) and linear math models (Type I; [73]), respectively, as the innate response of differential in environmental conditions. Although adult *N. tenuis* has exhibited a type-II functional response preying on *T. absoluta* eggs and *Ephestia kuehniella* eggs [77]. Michailidis et al. [78] found that *N. tenuis* showed a type-III functional response when preying on the eggs of *T. absoluta*. These type-III functional responses are characterized by the saturation of predators at high prey densities. It is known that the functional response of generalist predators can vary depending on prey species [79]. Some studies on the functional response of other mirid species, such as *Macrolophus basicornis*, *Engytatus varians*, and *Campyloneuropsis infumatus* in *T. absoluta* eggs, yielded results in a differentiated functional response under the same laboratory conditions [80]. *Engytatus varians* and *M. basicornis* showed a type-III functional response, while *C. infumatus* revealed a type-II functional response [80]. The functional-response model is probably due to prey biomass [81] or nutritional contributions [82]. Commonly, mathematical models are generally expressed in prey amount and not prey biomass, and the functional response may vary from one prey to another [83,84]. In the case of *N. tenuis*, the functional response may be affected by the host plant [85]. Also, prey density could influence the distribution of prey on leaflets, and low densities become more patchy than high densities. Consequently, the functional response of *N. tenuis* could be different considering the prey density level of *B. cockerelli*. However, this is the first study of the potential predator of *N. tenuis* on *B. cockerelli*. In this study, we observed that the functional responses during the laboratory and greenhouse studies might have been subjected to different environmental conditions that affected the predatory behavior of *N. tenuis* [86]. Under laboratory conditions, the temperature was 24.38 ± 0.06 °C, 85.78 ± 0.32% RH, and had a 5.7 ± 0.25 µmol/m^2^ s light intensity, while in the greenhouse the temperature was 24.98 ± 0.13 °C, 59.554 ± 0.36% RH, and had a light intensity of 13.89 ± 0.62 µmol/m^2^ s. The light intensity was 2.47 times higher in the greenhouse than under laboratory conditions in a growth chamber, with fluctuations during the day. In the greenhouse, the light intensity reached a maximum and minimum value of 0.36–19.04 µmol/m^2^ s. However, the greatest impact could have been due to temperature [86] because it fluctuated from 18 to 38 °C in the greenhouse. At 38 °C, we observed that *N. tenuis* was less active and remained in the coolest places, approaching the moist soil. Nevertheless, similar control levels of *B. cockerelli* populations were achieved with the 1.5:1 ratio of *N. tenuis*: *B. cockerelli* used in both conditions after ten days. There is a possibility that *N. tenuis* displayed a lethargic behavior under greenhouse conditions due to high temperatures. Despite *N. tenuis* origin, which is considered a species of subtropical temperatures, a study on temperatures in two subtropical *N. tenuis* strains reported that their survival and development were different for these two strains [87]. The two strains showed a thermotolerance correlated with their climatic origin. A low temperature of 15 °C was significantly more favorable for the thermal strain than the subtropical strain. However, the higher temperature was not significant for survival between the two strains, but they did observe torpor in their predatory activity [87].

In the present study, we showed that *N. tenuis* may survive just preying upon *B. cockerelli*. The predatory capacity of *N. tenuis* can be reduced depending on the environment. When confined to potato plants under field conditions during the present study, *N. tenuis* preyed on *B. cockerelli*, especially on nymphs. In field cages, two to three out of ten adults of *N. tenuis* survived for ten days on potato plants preying on *B. cockerelli*. Although short-term survival was low, *N. tenuis* adults managed to produce >1 nymph/cage, when enclosed on the potato plant and feeding on *B. cockerelli*. In the open-field trial with *N. tenuis* adult-controlled releases, these predatory nymphs were scarce on potato leaves. The results of the present study indicated that *N. tenuis* may regulate *B. cockerelli* populations under field conditions, because it was able to efficiently search for and feed on *B. cockerelli* eggs and nymphs. During the field trial, less than one (0.7–0.9) *N. tenuis* per plant released throughout the growing season was enough to significantly control *B. cockerelli*. In the Atlantic potato test in 2016, *N. tenuis* peaked at the start of the experiment on 9 February with 1.5 immature psyllids per leaf; this was the best treatment to reduce potato psyllid populations. After that, the effectiveness of treatment with *N. tenuis* releases was maintained at low prey numbers, below 2.5 psyllids per leaf. Biweekly releases of *N. tenuis* were effective and with fewer numbers of insecticide applications for the potato-growing season. But, when the *B. cockerelli* population was low, treatment with *N. tenuis* + RIP significantly decreased *B. cockerelli* populations. The alternate use of *N. tenuis* releases and RIP applications were more effective with low *B. cockerelli* populations than with predator releases alone. In addition, *N. tenuis* + RIP reduced the accumulated dose of insecticides at the end of the season. The standard program of insecticides is a sequence of blocks of insecticides with different modes of action, repeating weekly applications of the same insecticide. In the standard insecticide schedule, you have two to four applications of abamectin, followed by two of spirotetramat, and then four of spinetoram [88]. The low number of immature psyllids could have been the result of other factors, such as the lower susceptibility of cultivar FL-1867 to this psyllid coupled with a low seasonal infestation.

Regarding the incidence of ZC disease, we found a significant reduction in the percentage of infected tubers in cultivar FL 1867 under treatment with *N. tenuis* + RIP, but this difference vanished when the psyllids became scarce. Furthermore, our results indicated that the treatment with *N. tenuis* + RIP could indirectly increase the potato yield in both Atlantic and FL-1867 cultivars. It is suspected that the observed low percentage of tubers with ZC symptoms was due to a low population of immature and adult stages of *B. cockerelli* when FL-1867 potatoes were used in field trials.

Currently, there are no studies available regarding non-target (beneficial) insects that may be preyed upon by *N. tenuis*. However, it remains uncertain whether this mirid species could prey on beneficial insects in crops where releases are conducted or due to its natural occurrence. In potato crops, *N. tenuis* can function as a predator, targeting pest insects such as *B. cockerelli* and whiteflies, both prevalent pests in the area of study.

Since 2000, *B. cockerelli* has become the key pest insect of potatoes as the vector of ZC disease in South Texas [48,49]. *Bactericera cockerelli* developed resistance to insecticides in subtropical areas of Texas [55], and biological control has become highly relevant to the sustainable management of *B. cockerelli*. Before this study, a phytoseiid mite species (*Amblyseius largoensis*) had been reported as a biocontrol agent for *B. cockerelli* and excellently adapted to the prevailing conditions of potato cultivation in LRGV [89]. However, more biological control agents are needed to keep *B. cockerelli* populations under control. There is potential to use *N. tenuis* as a new biological agent of *B. cockerelli* in LRGV because *N. tenuis* has been established and adapted to South Texas since 2013 [11]. *Nesidiocoris tenuis* is present in tomato fields in LRGV, including at the start of the potato-growing season in the region [12].

Similar studies conducted with predator mirids such as *Engytatus nicotianae* (Koningsberger) (Hemiptera: Miridae) showed that they preyed on *B. cockerelli* under greenhouse conditions in New Zealand [84]. Also, the mirid *E. varians* (Distant) was an excellent predator of *B. cockerelli* both in the laboratory [90] and greenhouse [91]. Releases of the *Dicyphus hesperus* (Knight) in the greenhouse have resulted in the effective control of *B. cockerelli* in tomato production under protected structures [92]. However, the successful application of a biological control agent under natural conditions in most cases depends on the establishment and maintenance of the predator in a specific ecoregion, as shown by *N. tenuis* in the present study. *Nesidiocoris tenuis* showed a great preference for prey species such as *B. tabaci* or *T. absoluta*, but also has a selective reproduction strategy depending on the plant. For example, the intrinsic rate of *N. tenuis* in tomatoes becomes negative (−0.002) under laboratory conditions (25 ± 1 °C, 16:8 h L:D), while the 30-day intrinsic rate of *N. tenuis* reared on sesame was 4.0 under greenhouse conditions [60].

In this study, the results indicated that *N. tenuis* nymphs were present in potato crops without becoming a major pest. Also, the presence of *N. tenuis* nymphs in the *N. tenuis* + treatment showed a possible compatibility of the releases of *N. tenuis* with RIP. There is a delicate balance between predator activity and crop damage when *N. tenuis* is used as a biocontrol agent in some crops. In potatoes, we did not find evidence pointing to the fact that *N. tenuis* could become a pest, despite serial releases. However, it has been suggested that *N. tenuis* can become a pest of tomatoes given the prevalence and high abundance of this insect in tomato crops in the LRGV sub-ecoregion [12]. When *N. tenuis* is released to control whiteflies or other pests in tomato production systems, it can become a pest both in closed production systems and in the open field [12,93]. In tomato, a new world crop, *N. tenuis* can feed and negatively affect this plant, at the same time preying on *B. tabaci*, indicating a mirid zoophytophagous behavior. Knowledge about biological and phenological aspects of *N. tenuis* is limited in the LRGV, as well as phytophagy in wild or cultivated plants such as potatoes or other nightshade plants cultivated or wild species. In some predatory mirids, plant food is necessary for development and reproduction. Although, in some mirid species, plant feeding is optional and may result in earlier development with prominent reproduction [94]; in other species, plant feeding is still facultative and does not contribute to faster growth and/or higher reproduction but may result in survival when prey is absent. In the case of *N. tenuis*, sesame resulted in an appropriate host plant for its development and reproduction [95].

To our knowledge, this is the first report of *N. tenuis* preying upon *B. cockerelli* eggs and nymphs. Results of these studies showed that there was a novel interaction between the exotic *N. tenuis* predator and the native *B. cockerelli* prey, and this relationship could happen under controlled and natural environments. Our findings indicate a trend towards diminished *B. cockerelli* populations in the presence of *N. tenuis*, potentially requiring insecticide application to sustain *B. cockerelli* at desirable low population levels.

## 5. Conclusions

Considering all the information collected in this study about *N. tenuis*, a subtropical mirid species, we have shown that it can prey upon eggs and nymphs of *B. cockerelli.* In our studies, we found that control levels of *B. cockerelli* populations were achieved with the 1.5:1 ratio of *N. tenuis*: *B. cockerelli* used in tents and greenhouse conditions after ten days. In open-field trials conducted in this study, we found that less than one (0.7–0.9) *N. tenuis* per plant released throughout the growing season was enough to significantly control *B. cockerelli*. In addition, our studies have shown that releases of *N. tenuis* were compatible with standard reduced insecticide programs based on insecticides with different modes of action such as abamectin, spirotetramat, and spinetoram. Also, we predict that *N. tenuis* may have a positive impact on the production of potatoes and other solanaceous crops in the LRGV by reducing populations of *B. cockerelli*. *Nesidiocoris tenuis* nowadays occurs naturally in potatoes, tomato, pepper, and sesame in LRGV, and it can be compatible with reduced insecticide programs.

## Figures and Tables

**Table 1:** Reduced insecticide program (RIP) against *Bactericera cockerelli* and release dates, numbers, and ratios per plant of *Nesidiocoris tenuis* adults used in experimental potato crops under open-field studies during the 2016 and 2017 potato-growing seasons.

Insecticide *	Formulation **	Foliar Application					Field *Nesidiocoris tenuis* Releases		
		Quantity	Rate	Unit ***	Dates		No. Adults < 4-d (Sex Ratio 1:1)	Dates	
							20	4 February 2016	30 January 2017
							50	11 February 2016	5 February 2017
Spirotetramat	SC 2.00 LG	5	ZMA	1	12 February 2016	2 February 2017			
							50	19 February 2016	13 February 2017
Abamectin	EC 0.15 LG	8	ZMA	2	2 March 2016	17 February 2017			
							50	9 March 2016	24 February 2017
							20		13 March 2017
Pymetrozine	WG 50.00%	5.5	OMA	3	21 March 2016	3 March 2017			
							50	28 March 2016	24 March 2017
							50		7 April 2017
Spinetoram J and L	SC 1.00 LG	8	ZMA	4		16 March 2017			

* Insecticides were applied with an adjuvant (methylated seed oil + organo-silicone surfactant) at 0.25 PMV (% material vol to vol). ** SC: suspension concentrate; EC: emulsifiable concentrate; WG: water-dispersible granules. *** OMA: oz (dry) material/acre; ZMA: oz (fluid) material/acre.

## Data Availability

The datasets generated during and/or analyzed during the current study are available from the corresponding authors upon request.
